# Employee Net Promoter Score Links Nursing Satisfaction to Quality of Care Before and During the COVID‐19 Pandemic

**DOI:** 10.1155/jonm/1209322

**Published:** 2026-03-24

**Authors:** Jody S. Chou, Neel T. Shah, Amber Weiseth, Samuel R. Woodbury, Joyce K. Edmonds

**Affiliations:** ^1^ Departments of Epidemiology and Biostatistics, Harvard T.H. Chan School of Public Health, Boston, Massachusetts, USA, harvard.edu; ^2^ Department of Obstetrics and Gynecology, Western Pennsylvania Hospital of Allegheny Health Network, Pittsburgh, Pennsylvania, USA; ^3^ Delivery Decisions Initiative, Ariadne Labs, Boston, Massachusetts, USA; ^4^ Department of Nursing at Manning College of Nursing and Health Sciences, University of Massachusetts Boston, Boston, Massachusetts, USA, umb.edu

**Keywords:** job satisfaction, nursing, quality improvement, retention

## Abstract

**Aim:**

To measure the association between job satisfaction and perceived quality of care among nurses on labor and delivery (LD) units.

**Background:**

Nurses constitute the largest segment of the US healthcare workforce. Low job satisfaction is a critical factor in nurse turnover and quality of care.

**Methods:**

A web‐based survey was distributed across LD units in the United States. We used logistic regression to assess the association between job satisfaction, as measured by the employee net promoter score (eNPS), and perceived indicators of quality of care.

**Results:**

Among 1021 LD nurses who responded, those characterized as passive or detractors had greater odds of rating that the quality of care on their LD unit was fair or good rather than excellent (adjusted odds ratio [aOR] 3.45, 95% CI: 2.44–4.88 and aOR 6.58, 95% CI: 4.08–10.75, respectively, with both *p* < 0.0001), agreeing that nurses were spending less time with laboring patients suspected or confirmed to have COVID‐19 (aOR 1.42, 95% CI: 1.04–1.95, *p* = 0.0288; aOR 2.08, 95% CI: 1.33–3.28, *p* = 0.0014) and reporting that professional labor support was frequently or always missed during the pandemic (aOR 2.19, 95% CI: 1.49–3.20, *p* < 0.0001; aOR 1.82, 95% CI: 1.09–3.03, *p* = 0.0217) compared to respondents characterized as promoters.

**Conclusions:**

Higher nursing job satisfaction as measured by the eNPS is associated with higher perceived quality of care on the LD units.

**Implications for Nursing Management:**

eNPS is a one‐question survey that is easy to deliver, simple to interpret, and is associated with perceived quality of care. Nursing management can use it to track progress over time and understand reasons for job dissatisfaction on their units.

## 1. Introduction

The COVID‐19 pandemic had a devastating impact on the healthcare industry, with depression, anxiety, acute stress reaction, posttraumatic stress disorder, insomnia, and occupational burnout disproportionately affecting frontline staff [1]. Physicians and nurses report increasing rates of burnout and a heightened level of distrust with hospital administration [2]. Nurses, the largest professional group in healthcare and comprising 30% of total hospital employment, are pivotal to providing high‐quality care that is integral to patient safety and health outcomes [3]. Chronic understaffing of nurses during the pandemic posed risks to both worker and patient safety [4]. In a systematic review of nurses working in acute care hospital settings [5], the importance of governments, policymakers, and nursing groups in advocating and providing support for nurses both during and following the pandemic was detailed.

Nurse staffing on labor and delivery (LD) units is uniquely challenging, as it requires staffing levels that resemble intensive care units [6]. Plough et al. [7] identified four challenges to nursing management that are common to all LD units: (1) scheduling planned cases, (2) tracking patient flow, (3) monitoring bed and staff availability in the moment, and (4) adjusting bed and staff availability in the moment. Management decisions on LD units have shown to significantly impact clinical outcomes [8] and can be particularly crucial in maintaining a high standard of care during staff shortages in the midst of the COVID‐19 pandemic. Our team recently studied the roles and experiences of LD nurses during the pandemic and found that nearly half of the respondents reported changes in their roles and “picking up” additional responsibilities such as phlebotomy and housekeeping as a result of staffing changes [9]. In a study of a survey administered to registered nurse members of the Association of Women’s Health, Obstetric and Neonatal Nurses (AWHONN), Simpson et al. [10] described three main themes that emerged as a consequence of inadequate staffing: missed care, potential for failure to rescue, and job‐related stress and dissatisfaction.

In addition to adequate and effective staffing, individual nurses directly impact care delivery on LD units. Edmonds et al. [6] proposed measuring individual nursing performance by identifying nurse‐specific cesarean rates for low‐risk, nulliparous, term, singleton, and vertex presentation (NTSV) patients. In a retrospective cohort study of 72 LD nurses who attended 3031 births, nurses with higher individual NTSV cesarean rates were more likely than those with lower individual NTSV cesarean rates to attend cesarean births and thus suggest that LD nurses assigned to laboring patients may influence the likelihood of cesarean birth [11]. A subsequent study includes both LD nurses and physicians across three time points—admission, labor management, and delivery—and suggests that routine feedback on nurse‐level NTSV cesarean delivery rates could provide valuable insights and improve quality of care [12]. These findings contribute to emerging evidence that nurses affect birth outcomes.

To further examine the impact of individual nurses’ on quality of care on LD units, we focused on the influence of job satisfaction, as it is a known contributor to burnout. We evaluated several tools (Supporting Information 1) and chose to use the employee net promoter score (eNPS). eNPS is a one‐question survey that assesses how likely an individual is to recommend his/her institution to family and/or friends. The assumption is that intentions of promoting an institution suggest that the individual is content and satisfied with working there and would continue working at the institution. It was adapted from the widely used net promoter score (NPS) in the business sector for quantifying customer satisfaction and brand loyalty and may offer pragmatic advantages for administration as it is brief and helps characterize both level of job satisfaction and information about nursing retention. Our study examines job satisfaction among nurses in the United States using eNPS and assesses how it impacts perceived quality of care on LD units both prior to and during the COVID‐19 pandemic. Our three main research questions were as follows: (1) How satisfied are nurses with their jobs? (2) How is job satisfaction associated with quality of care? (3) How is job satisfaction associated with quality of care changes during the COVID‐19 pandemic?

## 2. Methods

### 2.1. Study Design

This is a cross‐sectional, descriptive survey study regarding LD nurse job satisfaction and correlation with quality of care prior to and during the COVID‐19 pandemic across the United States.

### 2.2. Ethical Approval

Prior to the start of data collection, ethical approval from the Institutional Review Board (IRB) was obtained from the Harvard T.H. Chan School of Public Health with research project reference no.: IRB19‐2108.

### 2.3. Participant Recruitment

Participants from the United States were recruited between July and August 2020 through multimodal online strategies including emails to professional societies such as AWHONN state sections and Perinatal Quality Collaborative and social media platforms including Facebook, LinkedIn, and Twitter. Certified registered nurses who were employed on LD units and provided direct patient care to intrapartum women were eligible to participate. The recruitment content provided participants with information to the study landing page. The study landing page provided participants with the purpose and goals of the study, eligibility criteria, and details of informed consent. After reviewing the informed consent, respondents consented to participate by clicking on the “agree” button.

### 2.4. Survey Instruments

Data for the analysis of the research questions were derived from the eNPS, the quality of care indicators, and the individual participant and hospital characteristics.

#### 2.4.1. eNPS

We measured job satisfaction among LD nurses using a one‐item question adopted from the eNPS tool: On a scale of 0–10, how likely are you to recommend the LD services at this hospital to your friends and family? A score of 0–6 classifies the individual as a detractor, 7–8 as passive, and 9–10 as a promoter. A single‐item instrument was chosen over a multi‐item instrument due to its ability to function as a low‐burden monitoring tool to track workforce sentiment, suitable for a clinical setting where time is limited, and frequent feedback is valuable.

#### 2.4.2. Quality of Care Indicators

An author‐derived survey was used to assess perceived quality of care on the LD floors both prior to and during the COVID‐19 pandemic. Although the first case of COVID‐19 in the United States was detected in January 2020, the pandemic was declared in March 2020. Participants’ interpretations of quality of care on the labor floor reference COVID‐19 as in March up until study collection time, which was August 2020, as compared to prior to the pandemic—likely before March 2020. The first item asked about quality of care prior to the pandemic (with ratings of excellent, good, fair, or poor), and the latter two items assessed quality of care changes during the pandemic. Changes in time spent at the bedside were assessed by asking participants to rate strongly disagree, disagree, agree, or strongly agree with the following statement: (1) nurses on my unit are spending less time at the bedside caring for laboring women who are suspected or confirmed COVID‐19 positive. The changes in missed professional labor support were assessed by asking participants to rate rarely, occasionally, frequently, or always to (2) how frequently has professional labor support (defined as continuous presence, providing emotional support, physical comfort, information for the woman and her family, advocacy, and anticipatory guidance and explanations of procedures) been missed (delayed, unfinished, or omitted) during labor and birth by the nursing staff (including you) within the past month.

#### 2.4.3. Individual Characteristics

Individual characteristics include age, gender, race, years practiced as a nurse, years practiced as an LD nurse, current job status (full‐time, part‐time, or per‐diem), highest completed level of nursing education, certified as a nurse midwife (Y/N), trained in Spinning Babies (Y/N), and usual shift.

#### 2.4.4. Hospital Characteristics

Hospital characteristics include location (urban, suburban, or rural), hospital type (academic medical center, community hospital, or other), ownership structure (for‐profit, private nonprofit, or government‐owned), American Nurses Credentialing Center (ANCC) Magnet designation (Y/N), teaching hospital (Y/N), annual delivery volume (< 1000, 1000–2499, or > 25,000 births per year), level of neonatal care (Level I–IV), admitting privileges for midwives (Y/N), and unit microculture score. The unit microculture (an aggregated average between 1 and 4 of an 8‐item survey, with 4 being the most supportive of vaginal birth delivery and positive culture) is one of six subscales from the Labor Culture Survey [13] (Supporting Information 2). The unit microculture subscale has been shown to be strongly associated with reduced rates of primary cesarean section [14].

### 2.5. Statistical Analysis

Descriptive analyses were performed to compare baseline characteristics across exposure groups. The chi‐square test was used for baseline characteristics that were categorical, and ANOVA was used for baseline characteristics that were continuous. Baseline characteristics were summarized using available‐case denominators. Participants with missing values for a given characteristic were excluded from only that item’s summary. No imputation was performed. Multivariate logistic regression was used to determine the strength of association between the exposure variable, job satisfaction quantified by eNPS, and outcome variables of interest—indicators of perceived quality of care. For statistical models that included variables with missing values, complete‐case analysis was utilized for which participants were dropped from the model if they had missing values. All data manipulation and analyses were conducted in RStudio 1.3. A *p* value cutoff of < 0.05 was used to determine statistical significance.

## 3. Results

A total of 1021 nurses from 43 states and the District of Columbia responded to the survey (Supporting Information 3). The median age group was between 35 and 44 years, the majority of the respondents were white (80.9%, *n* = 825) and identified as female (95.8%, *n* = 970). More than 75% of the respondents had a degree in Bachelor of Science and beyond (*n* = 792) and slightly lower than 25% had a Diploma in Nursing or an Associate Degree in Nursing (*n* = 229). Four hundred and thirty‐five participants (42.6%) have practiced as an LD nurse for more than 11 years, 220 participants (21.5%) for 6–10 years, and 366 (35.8%) for < 5 years. Staff nurses made up almost 70% of all participants (*n* = 707), with the remainder consisting of charge nurses, nurse managers, administrators, team leads, nurse educators, clinical nurse specialists, travel staff nurses, and others.

### 3.1. How Satisfied Are Nurses With Their Jobs?

Of our sampled individuals, 60.8% rated an eNPS of 9 or 10, signifying that they were promoter respondents and individuals who were satisfied with their jobs. Out of the remaining individuals, 26.2% scored an eNPS of 7 or 8 (passive respondents who were content but displayed neutral behaviors) and 13.0% had an eNPS between 0 and 6 (detractor respondents who were unhappy with their jobs) (Table 1).

**Table 1:** Demographics and hospital characteristics by the net promoter group.

Variables	Detractor	Passive	Promoter
	(*N* = 133)	(*N* = 267)	(*N* = 621)
Demographics			
Age^∗^ (%)			
18–34 years	47 (35.6)	83 (31.3)	197 (31.8)
35–54 years	69 (52.3)	150 (56.6)	322 (51.9)
55 years or older	16 (12.1)	32 (12.1)	101 (16.3)
Race^∗^ (%)			
American Indian or Alaska Native	23 (17.3)	10 (3.7)	28 (4.5)
Black or African American	11 (8.3)	16 (6.0)	18 (2.9)
White	87 (65.4)	201 (75.3)	537 (86.6)
Asian or Native Hawaiian or other Pacific Islander or prefer not to respond	12 (9.0)	40 (15.0)	37 (6.0)
Hispanic or Latina/Latino origin^∗^ (%)			
Yes	22 (16.5)	39 (14.6)	60 (9.7)
No or prefer not to respond	111 (83.5)	228 (85.4)	556 (90.3)
Highest level of nursing education (%)			
Diploma or associate	42 (31.6)	41 (15.4)	146 (23.5)
Bachelors, masters, DNP, PhD	91 (68.4)	226 (84.6)	475 (76.5)
Years of practice as a labor and delivery nurse			
< 5	65 (48.9)	107 (40.1)	194 (31.2)
6–10	27 (20.3)	64 (24.0)	129 (20.8)
> 11	41 (30.8)	96 (36.0)	298 (48.0)
Main position (%)			
Staff nurse	87 (65.4)	175 (65.5)	445 (71.7)
Other	10 (7.5)	9 (3.4)	22 (3.5)
Charge nurse, CNS, educator, manager	36 (27.1)	83 (31.1)	154 (24.8)
Job status (%)			
Full‐time	115 (86.5)	208 (77.9)	459 (73.9)
Part‐time	10 (7.5)	40 (15.0)	113 (18.2)
Per‐diem	8 (6.0)	19 (7.1)	49 (7.9)
Hospital characteristics			
Hospital location (%)			
Urban	71 (53.4)	134 (50.2)	274 (44.1)
Suburban	46 (34.6)	108 (40.4)	273 (44.0)
Rural	16 (12.0)	25 (9.4)	74 (11.9)
Type of hospital^∗^ (%)			
Academic medical center	55 (41.4)	118 (44.4)	221 (35.6)
Community hospital	78 (58.6)	148 (55.6)	400 (64.4)
Ownership structure of hospital (%)			
For profit	30 (22.6)	54 (20.2)	124 (20.0)
Private nonprofit	66 (49.6)	132 (49.4)	339 (54.6)
Government‐owned	23 (17.3)	42 (15.7)	47 (7.6)
Uncertain	14 (10.5)	39 (14.6)	111 (17.9)
ANCC Magnet designation hospital (%)			
Yes	63 (47.4)	130 (48.7)	293 (47.2)
No	70 (52.6)	137 (51.3)	328 (52.8)
Teaching hospital^∗^ (%)			
Yes	81 (61.4)	160 (61.1)	355 (59.0)
No	51 (38.6)	102 (38.9)	247 (41.0)
Hospital annual delivery volume^∗^ (%)			
< 1000 births per year	64 (48.5)	92 (35.2)	190 (31.0)
1000–2499 births per year	37 (28.0)	75 (28.7)	154 (25.1)
> 2500 births per year	31 (23.5)	94 (36.0)	269 (43.9)
Hospital level of neonatal care (%)			
Level I, well newborn nursery	23 (17.3)	41 (15.4)	97 (15.6)
Level II, special care nursery	40 (30.1)	81 (30.3)	158 (25.4)
Level III, neonatal intensive care unit	49 (36.8)	110 (41.2)	281 (45.2)
Level IV, regional neonatal intensive care unit	19 (14.3)	33 (12.4)	79 (12.7)
Uncertain	2 (1.5)	2 (0.7)	6 (1.0)
Midwives admitting privileges (%)			
Yes	79 (59.4)	155 (58.1)	372 (59.9)
No	54 (40.6)	112 (41.9)	249 (40.1)
Unit microculture score (points)	2.43 ± 0.51	2.72 ± 0.36	2.94 ± 0.37

*Note:* Missing data: ^∗^4 age, 1 race, 5 Hispanic or Latina/Latino status, 1 hospital type. Uncertain responses: 25 on teaching hospital status and 15 on birthing volume.

Statistically significant differences were observed between baseline characteristics across the three net promoter groups (promoter, passive, and detractor) for race, Hispanic or Latina/Latino origin, highest level of nursing education, years practiced as an LD nurse, and job status. Differences in race distribution were noted with the promoter group having more white respondents (86.6%) than the passive group (75.3%) and the detractor group (65.4%). The proportion of both American Indian/Alaska Native and Hispanic/Latino/Latina origin was greatest in the detractor group, with 17.3% and 16.5%, respectively. Passive respondents had the highest percentage of a Bachelor’s degree or higher (84.6%), and detractor respondents had the highest percentage of either a Diploma or Associate Degree in Nursing (31.6%). The promoter group had the highest proportion of individuals who practiced as an LD nurse for > 11 years (48%), and the detractor group had the highest proportion of individuals who worked as an LD nurse for < 5 years (48.9%). All three groups (promoter, passive, and detractor) were made up of a majority of full‐time employees, with the proportion of part‐time employees in the passive and promoter groups twice as high or higher as in the detractor group.

The proportion of respondents working in community hospitals was the highest for the promoter group (64.4%), whereas the proportion of respondents working in academic medical centers was the highest in the detractor group (41.4%). The proportion of respondents working in hospitals with the highest annual delivery volumes (> 2500 births per year) was the greatest among the promoter group (43.9%), then the passive group (36.0%), and then the detractor group (23.5%). The unit microculture score was highest in the promoter group (2.94), then the passive group (2.72), and then the detractor group (2.43). There were no statistically significant differences in the hospital location, ANCC Magnet designation, teaching hospital, level of neonatal care, and midwives’ admitting privileges of hospitals employing detractor, passive, and promoter individuals.

### 3.2. How Is Job Satisfaction Associated With Quality of Care?

Detractor and passive respondents were more likely to rate the quality of care on their LD unit as subpar (compared with promoters). The odds of detractor respondents rating the QoC on their LD unit as fair or good (vs. excellent) were 6.6 times those of the promoter respondents (aOR 6.58, 95% CI: 4.08–10.75, *p* < 0.0001) (Table 2). The odds of passive respondents rating the QoC on their LD unit as fair or good (vs. excellent) were 3.5 times those of the promoter respondents (aOR 3.45, 95% CI: 2.44–4.88, *p* < 0.0001). Our model adjusted for age, highest level of nursing, years as an LD nurse, main position, type of hospital, ANCC Magnet designation hospital, teaching hospital, hospital annual delivery volume, and unit microculture.

**Table 2:** Crude and adjusted association between the net promoter group and good/fair quality of care on the unit.

Variable	Crude			Adjusted^∗^		
	OR	95% CI	*p* value	OR	95% CI	*p* value
Net promoter group						
Promoter	1.00	—	—	1.00	—	—
Passive	4.47	3.23, 6.20	< 0.0001	3.45	2.44, 4.88	< 0.0001
Detractor	11.12	7.26, 17.30	< 0.0001	6.58	4.08, 10.75	< 0.0001

*Note:* Net promoter groups: promoter (employee net promoter score [eNPS] of 9 or 10, satisfied with job and would recommend to family members/friends), passive (eNPS of 7 or 8, content with job and display neutral behaviors), or detractor (eNPS of 0–6, unhappy and unsatisfied with job and would not recommend to family members/friends). Referent group of excellent quality of care on the unit compared to good/fair quality of care on the unit.

^∗^Adjusted for age, highest level of nursing, years as a LD nurse, main position, type of hospital, ANCC Magnet designation hospital, teaching hospital, hospital annual delivery volume, and unit microculture.

### 3.3. How Is Job Satisfaction Associated With Quality of Care Changes During the COVID‐19 Pandemic?

Among respondents, 40.5% reported that there was rarely any missed professional labor support on their units, 37% reported that there was occasionally missed labor support, 18.6% reported that support was frequently missed, and 3.9% reported that there was always missed labor support during the COVID‐19 pandemic. Compared with promoter respondents, detractors and passive respondents both had higher odds of rating that professional labor support was frequently or always missed during the pandemic (aOR 1.82, 95% CI: 1.09–3.03, *p* = 0.0217; aOR 2.19, 95% CI: 1.50–3.20, *p* < 0.0001) (Table 3). Furthermore, 45.1% of the participants perceived that nurses on their units were spending less time at the bedside caring for laboring women suspected or confirmed to be COVID‐19 positive, whereas 54.9% did not perceive that time at the bedside was reduced. Respondents categorized as detractors had higher odds of rating that nurses were spending less time with laboring patients suspected or confirmed to have COVID‐19 (aOR 2.08, 95% CI: 1.33–3.28, *p* = 0.0014), after controlling for potential confounders. Respondents categorized as passive also had higher odds of rating that nurses were spending less time with laboring patients suspected or confirmed to have COVID‐19 (aOR 1.42, 95% CI: 1.04–1.95, *p* = 0.0288) than respondents categorized as promoters (Table 4).

**Table 3:** Crude and adjusted association between the net promoter group and odds of frequently/always missed professional labor support during the COVID 19 pandemic.

Variable	Crude			Adjusted^∗^		
	OR	95% CI	*p* value	OR	95% CI	*p* value
Net promoter group						
Promoter	1.00	—	—	1.00	—	—
Passive	2.65	1.89, 3.73	< 0.0001	2.19	1.49, 3.20	< 0.0001
Detractor	2.67	1.74, 4.06	< 0.0001	1.82	1.09, 3.03	0.0217

^∗^Adjusted for age, highest level of nursing, years as a LD nurse, main position, type of hospital, ANCC Magnet designation hospital, teaching hospital, hospital annual delivery volume, and unit microculture.

**Table 4:** Crude and adjusted association between the net promoter group and odds of spending less time at bedside caring for laboring women who are suspected or confirmed to be COVID‐19 positive.

Variable	Crude			Adjusted^∗^		
	OR	95% CI	*p* value	OR	95% CI	*p* value
Net promoter group						
Promoter	1.00	—	—	1.00	—	—
Passive	1.63	1.22, 2.20	0.0011	1.42	1.04, 1.95	0.0288
Detractor	2.58	1.75, 3.83	< 0.0001	2.08	1.33, 3.28	0.0014

^∗^Adjusted for age, highest level of nursing, years as a LD nurse, main position, type of hospital, ANCC Magnet designation hospital, teaching hospital, hospital annual delivery volume, and unit microculture.

## 4. Discussion

This study assessed the association between job satisfaction in healthcare workers using eNPS and the perceived quality of care delivered. We found that on the LD unit, nursing job satisfaction is associated with higher perceived quality of care provided both prior to and during the COVID‐19 pandemic. The detractors or passive respondents in our study had higher odds of rating that the quality of care was lower on their LD unit than promoter respondents. These findings indicate the importance of understanding the reasons for job dissatisfaction within an institution and investing in resources to shift detractor and passive employees toward being promoter employees.

Our findings of a positive association between job satisfaction and perceived quality of care are in line with results from other studies that also evaluated this linkage [15–19]. While these other approaches quantify job satisfaction by directly asking whether nurses are satisfied or dissatisfied with their jobs, our approach uniquely also captures how likely an employee is to refer services at his/her institution to friends and family through the use of the eNPS tool. This is a noticeable strength in our study design as it captures worker engagement and turnover intention, which are both key factors important to nurse management as they relate to a unit’s turnover rate [20–25].

This study has several limitations. First, our assessment of perceived quality of care relied on author‐derived survey questions rather than validated instruments, which may limit the validity and comparability of our findings. Second, because participant data were deidentified due to COVID‐related restrictions, we could not link responses to objective clinical outcomes such as nurse‐level NTSV cesarean rates or patient complications. Consequently, our results reflect perceptions of care quality rather than direct measures of clinical performance.

Third, the use of a convenience sample—primarily from Massachusetts, Oklahoma, California, and New Jersey—limits generalizability, as findings may not represent LD units nationwide. We also recruited participants through multiple open channels, therefore unable to calculate the traditional response rate, which limits our ability to assess nonresponse bias. Although we did adjust for covariates such as age, years as an LD nurse, unit microculture, and hospital annual delivery volume, residual confounding may still exist such as staffing and region practice patterns. Additionally, while the eNPS provides a simple and scalable measure of engagement, it may not fully capture the multifaceted nature of job satisfaction, and unmeasured factors could confound the observed associations. Finally, our cross‐sectional design precludes causal inference between job satisfaction and perceived care quality.

Despite these limitations, our study demonstrates the potential utility of the eNPS as a practical workforce engagement tool for nurse management and hospital leadership. With refinement and broader implementation, the eNPS could serve not only as a measure of staff sentiment but also as an early indicator of organizational health and perceived care quality over time.

### 4.1. Implications for Nursing Management

Findings from this study have direct implications for nurse managers who are committed to improving job satisfaction among their nursing staff and promoting higher quality of care to patients served by their units. More specifically, eNPS is a one‐question tool that is easy to administer and simple to interpret. It not only provides management with a percentage breakdown of the proportion of nurses who are promoters, passive, or detractor employees on their units, but it also provides a single number (e.g., institutional eNPS) that can be followed over time to assess progress in the long term. Furthermore, we also encourage nurse managers to ask the follow‐up question of what can be changed in order for them to improve their scores to a 9 or 10. This will help generate feedback that is specific to the unit and provide actionable items that management can adapt to improve job satisfaction and retention rate. These qualitative suggestions are all the more important, as the nursing profession endures a heightened level of burnout and mental health traumas from the COVID‐19 pandemic.

## 5. Conclusions

This study contributes to the growing body of evidence that higher nursing job satisfaction is associated with a higher quality of care. This was evident in our study sample both prior to and during the COVID‐19 pandemic. It also underscores the importance of tracking job satisfaction long‐term in order to assist management with improving work environments and reducing turnover rates as the profession continues to face challenges and address residual impacts from the COVID‐19 pandemic.

## Funding

This project was supported by a grant from the Controlled Risk Insurance Company/Risk Management Foundation of the Harvard Medical Institutions. It was also partially supported by the Health Resources and Services Administration (HRSA) of the U.S. Department of Health and Human Services (HHS) under grant T76MC00001 and entitled Training Grant in Maternal and Child Health.

## Conflicts of Interest

The authors declare no conflicts of interest.

## Supporting Information

Supporting Information 1: Tools for quantifying nurse job satisfaction.

Supporting Information 2: Unit microculture survey.

Supporting Information 3: Distribution of survey respondents across the United States.

## Supporting information


**Supporting Information** Additional supporting information can be found online in the Supporting Information section.

## Data Availability

The data that support the findings of this study are available upon request from the corresponding author. The data are not publicly available due to privacy or ethical restrictions.
