# Supplementary material for: Employee Net Promoter Score Links Nursing Satisfaction to Quality of Care Before and During the COVID‐19 Pandemic
Source: J Nurs Manag. 2026 Mar 24;2026:1209322. doi: 10.1155/jonm/1209322 (PMC13140922; doi:10.1155/jonm/1209322)
Supplement: Supplementary file 1 — Supporting Information Additional supporting information can be found online in the Supporting Information section. [file JONM-2026-1209322-s001.docx]

**Supplementary materials**

Supplement 1: Tools for Quantifying Nurse Job Satisfaction

(1) The Index of Work Satisfaction (IWS): characterizes overall nurse satisfaction as a weighted composite of the following six categories: pay, autonomy, task, organizational policies/ decision making, professional status, and nurse-nurse interaction [1].

(2) The RN Survey with Practice Environment Scale © (PES ©): developed by the nursing quality improvement program initially formed by the American Nurses Association (ANA) called The National Database of Nursing Quality Indicators ® (NDNQI ®); incorporates an adapted version of the IWS and additional existing surveys to form 9 total domains: nurse participation, nursing foundations for quality-of-care, nurse manager, staffing and resource adequacy, nurse-physician relations, nurse-nurse interaction, and job enjoyment [2].

(3) National Sample Survey of Registered Nurses (NSSRN): conducted by the United States Census Bureau on behalf of the Health Resources and Services Administration, includes following question for assessing job satisfaction: “How satisfied are you with your principal job, or most recent job if you are not now working?” with the options “extremely satisfied, moderately satisfied, neither satisfied nor dissatisfied, moderately dissatisfied, extremely dissatisfied, and neither currently nor previously employed” [3].

(4) employee Net Promoter Score (eNPS): an adoption of the one-question instrument (ie Net Promoter Score) that was widely used by companies to measure customer loyalty and drive business growth, states “On a scale of 0-10, how likely are you to recommend the labor and delivery services at this hospital to your friends and family?” [4].

Supplement 2: Unit microculture survey

1. In my hospital, doulas who accompany women in labor are welcomed into the labor support team
2. In my L&D unit, labor nurses are encouraged and supported to spend the majority of their time in the room with the patient throughout her labor
3. In my L&D unit, provider work flow considerations affect medical interventions in labor
4. Most of my patients have sufficient knowledge about vaginal and cesarean birth to make informed choices
5. Our L&D staff are skilled at providing effective labor coping strategies
6. Staff on my L&D unit support the laboring women’s informed choices, values, and preferences
7. The culture of my L&D unit supports vaginal birth and discourages overuse of cesarean sections
8. There are too many cesarean births performed in my L&D unit

Note: One of six subscales from the Labor Culture Survey, calculated as an aggregated average between 1 to 4 of the 8-item survey, 4 being the most supportive of vaginal birth delivery and positive culture

Supplement 3: Distribution of survey respondents across the United States

**
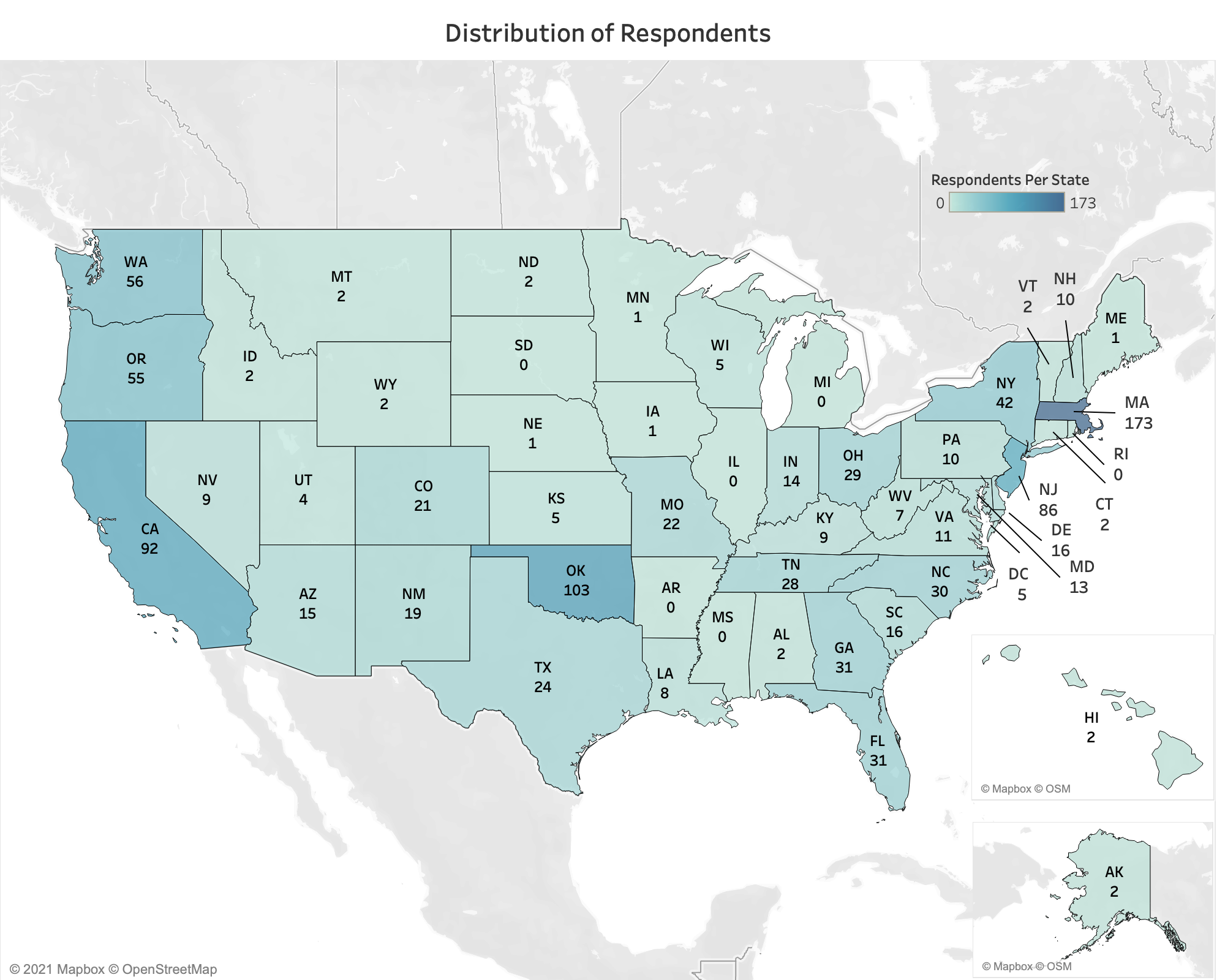
**

References

1. Stamps, P. L., & Piedmonte, E.B. (1986). Nurses and work satisfaction: An index for measurement. . Ann Arbor, Mich: Health Administration Press Perspectives.
2. Montalvo, I. (2007). The National Database of Nursing Quality Indicators™ (NDNQI®). Online Journal of Issues in Nursing, 12. https://doi.org/10.3912/OJIN.Vol12No03Man02
3. U.S. Department of Health and Human Services, H. R. a. S. A. (2010). The Reigstered Nurse Population. Findings from the 2008 National Sample Survey of Registered Nurses. https://data.hrsa.gov/DataDownload/NSSRN/GeneralPUF08/rnsurveyfinal.pdf
4. Reichheld, F. F. (2003). The One Number You Need to Grow. Harvard Business Review. https://hbr.org/2003/12/the-one-number-you-need-to-grow
